# RETRACTED: Locri et al. Protective Efficacy of a Dietary Supplement Based on Forskolin, Homotaurine, Spearmint Extract, and Group B Vitamins in a Mouse Model of Optic Nerve Injury. *Nutrients* 2019, *11*, 2931

**DOI:** 10.3390/nu18132153

**Published:** 2026-07-03

**Authors:** Filippo Locri, Maurizio Cammalleri, Massimo Dal Monte, Dario Rusciano, Paola Bagnoli

**Affiliations:** 1 Department of Biology, University of Pisa, Via San Zeno, 31, 56127 Pisa, Italy; filippo.locri1@gmail.com (F.L.); maurizio.cammalleri@unipi.it (M.C.); 2 Interdepartmental Research Center Nutrafood “Nutraceuticals and Food for Health”, University of Pisa, Via del Borghetto 80, 56124 Pisa, Italy; 3 Sooft Italia SpA, Contrada Molino 17, 63833 Montegiorgio (FM), Italy; dario.rusciano@sooft.it

The Journal retracts the article “Protective Efficacy of a Dietary Supplement Based on Forskolin, Homotaurine, Spearmint Extract, and Group B Vitamins in a Mouse Model of Optic Nerve Injury” [1], cited above.

Following publication, concerns were brought to the attention of the Editorial Office regarding the integrity of the figures presented in the article [1].

In accordance with standard journal procedures, the Editorial Office and Editorial Board conducted an investigation that confirmed concerns in Figure 3, including inappropriate image editing and duplication between panels presented in this figure and material from two different published articles [2,3] presenting different experimental conditions. Although the authors cooperated with the investigation and provided supporting materials, the Editorial Board concluded that the concerns could not be satisfactorily resolved based on the available information. As a consequence, the Editorial Bord lost confidence in the reliability of the findings and has decided to retract this publication in accordance with MDPI’s retraction policy.

This retraction was approved by the Editor-in-Chief of *Nutrients*.

The authors have been informed of this retraction and have indicated their agreement with this decision.
